# Survey of Blood Groups *DEA 1, DEA 4, DEA 5, Dal*, and *Kai 1/Kai 2* in Different Canine Breeds From a Diagnostic Laboratory in Germany

**DOI:** 10.3389/fvets.2020.00085

**Published:** 2020-02-28

**Authors:** Anne K. Ebelt, Sonja Fuchs, Corinna Weber, Elisabeth Müller, Urs Giger

**Affiliations:** ^1^Laboklin GmbH&Co KG, Kissingen, Germany; ^2^PennGen Laboratory, University of Pennsylvania, Philadelphia, PA, United States

**Keywords:** blood type, blood compatibility, transfusion reaction, polymorphism, alloantibodies, dog

## Abstract

More than twelve blood group systems have been described in dogs, but little is known about their distribution frequencies within breed populations. Here, we report on an extensive typing survey carried out using available reagents and either established or new clinical kits in purebred dogs from Germany. Leftover anticoagulated blood samples were examined using an immunochromatographic strip method for *DEA 1*, a gel column technique for *Dal* and *Kai 1/2*, and new card agglutination tests for *DEA 4* and *DEA 5* (which were partially compared with the gel column technique). Monoclonal antibodies were used for *DEA 1* and *Kai 1/2* typing, and polyclonal antibodies were used for all other types. Among the 206 dogs, 59.2% were *DEA 1*+, 100% *DEA 4*+, 9% (Card)/11% (Gel) *DEA 5*+, 89.3% *Dal*+, 96.6% *Kai 1*+, and 2.9% *Kai 2*+. None of the dogs were *Kai 1*+*/2*+, and only one was *Kai 1–/2–*. *Dal–* dogs were found in several breeds. Erythrocytes from most *DEA 1*+ dogs bound strongly on the strips. The agglutination reactions for *DEA 5* on the new card tests were generally less than those on the gel column. The blood group pattern *DEA 4*+, *DEA 5–, Dal*+, *Kai 1*+*/2–* and either *DEA 1*+ or *DEA 1–* was found among 80% of the dogs. In this first extensive blood typing survey of purebred dogs from Europe, the proportions of positive and negative blood types were similar to those found in the United States and, for *DEA 1*, were also similar to those from other European countries, with considerable breed variation in blood types. The newer typing techniques seem to work well and will likely be useful for detecting and preventing specific blood type incompatibilities in the clinic.

## Introduction

Canine blood group systems are defined by the expression of various antigens on the surface of red blood cells (RBCs), with different individuals either missing a specific antigen (negative blood type) or expressing it to varying degrees (positive blood type) (1, 2). Because individuals lacking the expression of a certain antigen may develop transfusion reactions following subsequent transfusions due to naturally occurring or induced alloantibodies, accurate typing of canine blood types is essential to prevent potentially fatal blood incompatibility reactions in clinics (3–6).

While canine blood groups have been studied for more than half a century (7, 8), none have been characterized at the biochemical and/or molecular genetic level. Instead, canine blood group systems, which now number more than a dozen, have been identified utilizing antisera from previously transfused dogs and/or the specifically generated monoclonal antibodies (1, 9). An international committee meeting in the 1970s designated the initially identified blood group systems as Dog Erythrocyte Antigen—*DEA* (10). Since then, additional blood group systems have been proposed, including *Dal* (11), *Kai 1* and *Kai 2* (12), and other currently unclassified blood groups (9). Moreover, additional blood group systems are suspected to exist based upon incompatible major crossmatch test results observed in previously transfused dogs (13).

While some blood group systems are well accepted, there is still some controversy regarding the presence of clinically important naturally occurring alloantibodies against *DEA 3, DEA 5*, and *DEA 7* (2, 7, 14, 15). However, there have been no acute hemolytic transfusion reactions reported in dogs in clinical settings, and similarly, no neonatal isoerythrolysis has been reported in puppies unless the dogs have been previously transfused (16, 17). Therefore, the presence and clinical importance of any naturally occurring alloantibodies are questioned.

The blood group system *DEA 1* is clinically considered most important due to the strong *DEA 1* antigenicity and the fact that *DEA 1*+ and *DEA 1–* dogs are found in relatively equal proportions (14, 18). The *DEA 1* blood group system was originally proposed to have several subtypes: *DEA 1.1 (A1), DEA 1.2 (A2)*, and *DEA 1.3 (A3)* (7, 8). However, recent studies indicate that the *DEA 1* antigen(s) can be recognized by a single monoclonal antibody, with the antigen(s) variably expressed from weakly to moderately to strongly positive (19). The level of expression of *DEA 1* antigen(s) is genetically determined and does not change over time or during storage of blood (19, 20).

Blood typing methods have evolved from tube and card tests to immunochromatographic strip, cartridge, and flow cytometry assays, but additional typing kits for other blood types would be desirable (1, 21–24). Several limited surveys have been performed for *DEA 1*, and a few have assessed other blood types in North America and Europe (25–35), but there have been no comprehensive surveys carried out on a canine population typing for many blood groups.

The purpose of this study was to determine the prevalence of several blood types in dogs using available reagents and kits at a diagnostic reference laboratory in Germany. We also determined the degree of *DEA 1* antigen expression and introduced two novel typing kits, i.e., agglutination cards for *DEA 4* and *DEA 5*. Finally, we compared our typing results with studies from the USA and other countries. This represents the first extended blood typing survey in purebred dogs from Europe.

## Materials and Methods

### Dogs and Blood Samples

Leftover ethylene-diaminetetraacetic acid (EDTA)-anticoagulated blood samples from purebred dogs that had been submitted for routine diagnostic work to Laboklin GmbH& Co KG, Kissingen, Germany between December 2018 and October 2019 were used for this survey. Of a total of 206 samples, the majority (188) originated from Germany, with 18 samples from neighboring or other European countries (Czech Republic, Denmark, Finland, Italy, Luxembourg, Sweden, and Norway). The approval to use leftover samples for research was received from the Government IACUC in Bayern, Germany. Only leftover samples with of least 1 ml EDTA blood that was less than 1 week old and had been stored at 4–8°C prior to analysis were included. Samples from several dog breeds, classified based on the Fédération Cynologique Internationale (FCI^1^), were selected based upon prior studies (11, 12, 27, 29, 36, 37) and our own early results of their varied blood group antigen expression. Samples from known related dogs or repeat samples from the same dog were excluded. Data on breed, gender, and age as well as geographic region were obtained. None of the samples showed any auto-agglutination in card and gel tests. All results were photographically captured.

### Blood Typing Assays

#### *DEA 1* Typing by Immunochromatographic Strip

**Figure 1:**
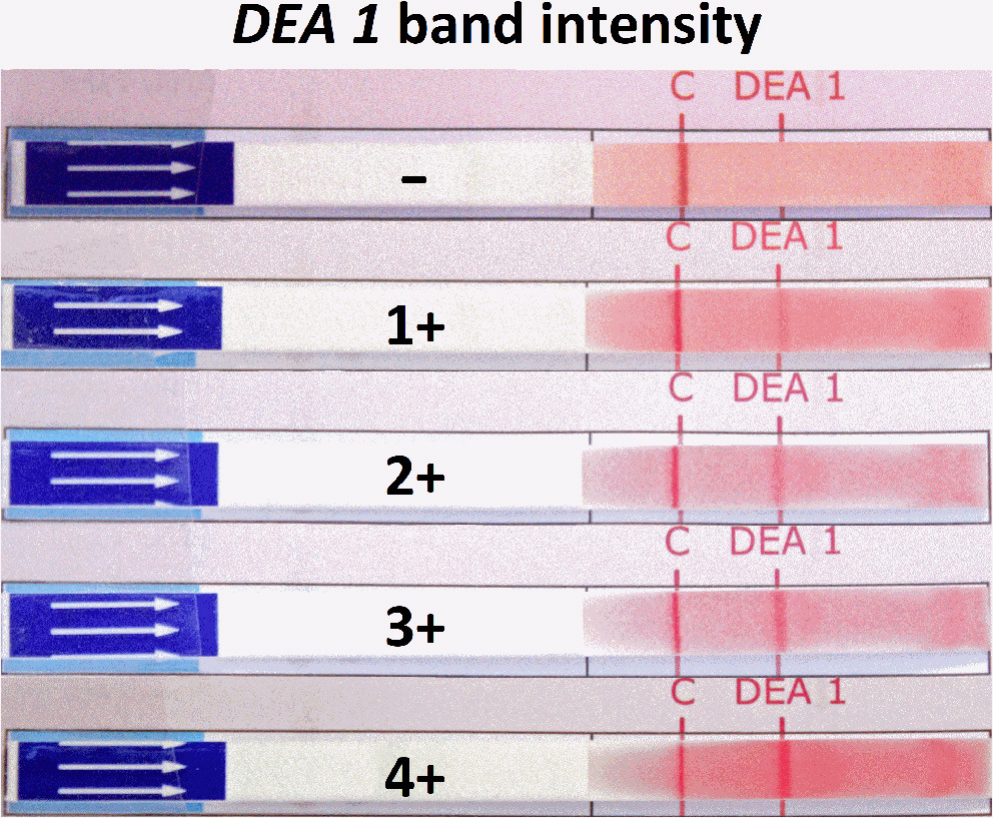
*DEA 1* typing results on immunochromatographic strips from 206 dogs. The varied binding intensities to a monoclonal anti-*DEA 1* antibody at the *DEA 1* position on the strip were graded from – (no band, negative) to 1+ to 4+ (band, positive). Red blood cells in suspension migrated in the membrane; the C (control) band had to show for it to be a valid test, while a variably strong *DEA 1* band was visible in some cases.

An immunochromatographic strip test (Strip, Lab Test *DEA 1*, Alvedia, Limonest, France) with a murine monoclonal anti-*DEA 1* alloantibody was used according to the manufacturer's instructions and as previously described (19, 27). The strength of the *DEA 1* band was semi-quantitatively assessed by visually comparing the control (anti-glycophorin antibody) band to the *DEA 1* band and grading it from – to 4+ (Figure 1), as previously described (27, 38).

#### *DEA 4* and *DEA 5* Typing by Agglutination Cards

Newly introduced agglutination cards (Card, RapidVet-H *DEA 4* Agglutination Card Test, and *DEA 5* Card RapidVet- H *DEA 5* Agglutination Card Test, DMS, Flemington, NJ, USA) with polyclonal anti-*DEA 4* or anti-*DEA 5* typing reagents (antisera) were used for this survey according to the manufacturer's instructions, with minor modifications. Prior to utilizing these cards in this survey, they were validated with *DEA 4*+ and *DEA 4–* RBCs as well as with *DEA 5*+ and *DEA 5–* RBCs, respectively, by the manufacturer.

**Figure 2:**
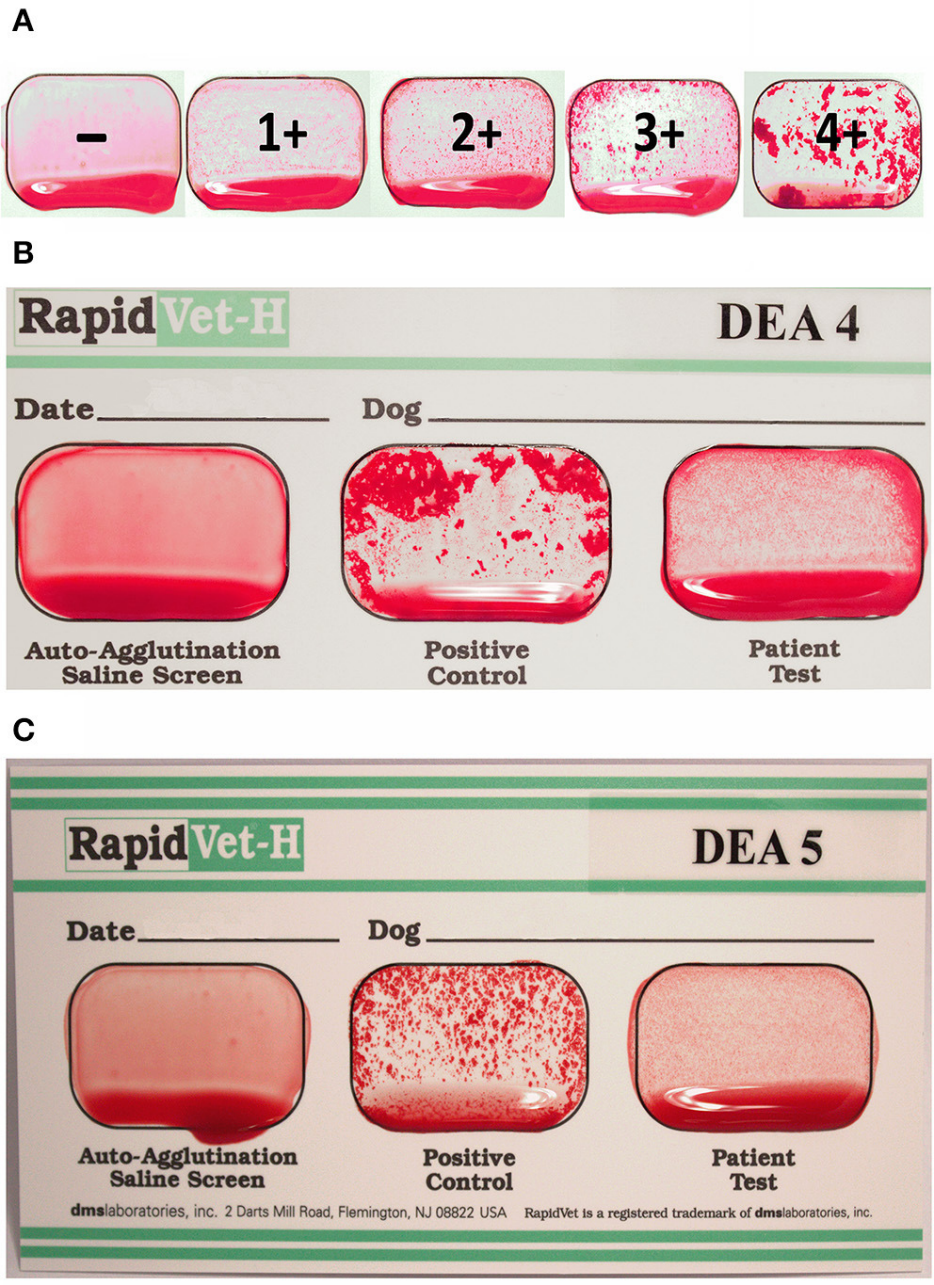
New agglutination cards for *DEA 4* and *DEA 5* typing of dogs. **(A)** Grading of the agglutination reaction strength from – to 4+. **(B)** *DEA 4*+ and **(C)** *DEA 5*+ showing weak agglutination reactions (1+). In each of the three wells of a card, 40 μl (*DEA 4*) or 80 μl (*DEA 5*) of buffer and 50 μl of EDTA blood from one dog was added and gently mixed. The well for “Auto-Agglutination-Saline Screen” and “Positive Control” must be negative and positive, respectively, to interpret the typing results. Depending on whether there is agglutination in the “Patient Test” well, the dog is considered positive or negative.

While the manufacturer's instructions suggested the use of 40 μl of diluent for *DEA 4* typing, very weak resulting agglutination reactions prompted us to add an additional 40 μl of diluent after the initial reading to achieve a total of 80 μl of diluent (identical to the volume in the protocol for *DEA 5* typing). This was routinely performed for 191 of 206 samples after the occurrence of weak agglutination reactions was observed with some of the first blood samples. The lack of and degree of an agglutination reaction was recorded from – to 4+ (Figure 2A).

Briefly, 50 μl of EDTA blood was added to 40 μl of diluent for *DEA 4* typing or 80 μl of diluent for *DEA 5* typing in each of the three wells on one card. Using the wooden stirrer provided, the reagents adhering to both the positive control and the patient well surface were rubbed off (no reagent in auto-agglutination control) and mixed to cover the entire well. The cards were gently rocked for 30 s and then examined for agglutination reactions, first while still rocking and once again after slightly tilting the card to allow excess blood to run to the bottom of each well.

If there were no visible agglutinations in the auto-agglutination well and a visible agglutination reaction in the positive control well, the assay was considered valid. Depending on the presence or absence of an agglutination reaction in the patient test well, the dog is considered weakly (1+) to strongly positive (3+/4+) or negative (–) for the respective blood group (Figures 2B,C).

Any degree of agglutination was considered a positive typing result. While the agglutination card testing was performed with all samples, gel column assay (see below) was also performed on some samples (*n* = 158) using the same *anti-DEA 4* and *anti-DEA 5* alloantibodies.

#### *DEA 4* and *DEA 5, Dal, Kai 1*, and *Kai 2* Typing by a Gel Column Technique

**Figure 3:**
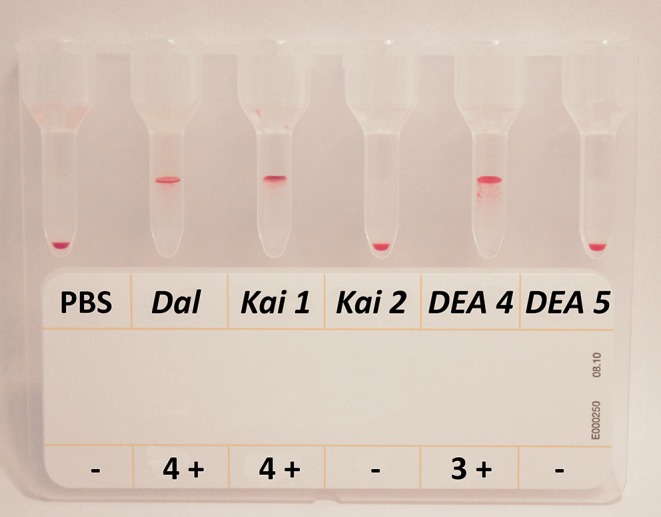
Gel column typing results for *Dal, Kai 1, Kai 2, DEA 4*, and *DEA 5* from one dog. The blood sample from this dog shows a typical typing pattern: 4+ agglutination reactions for *Dal* and *Kai 1*, no agglutination for *Kai 2*, a 3+ agglutination reaction for *DEA 4*, and no agglutination for *DEA 5*. Red blood cells at the top (4+) to within the gel mean positive agglutination reactions. PBS, phosphate-buffered saline, serves as auto-agglutination control.

A gel column technique (Gel, using BioRad ID-Cards, NaCl, Enzyme Test and Cold Agglutinins, DiaMed, Cressier, Switzerland) was used for *DEA 4* and *DEA 5, Dal, Kai 1*, and *Kai 2* typing, similar to our previous studies (24, 27, 29, 39). Briefly, EDTA blood samples were washed three times and diluted to a 2% RBC suspension using phosphate-buffered saline. Canine antisera containing *anti-DEA 4* or *anti-DEA 5* polyclonal alloantibodies from previously sensitized dogs (Animal Blood Resources International, Michigan, USA, provided by DMS Laboratories Inc.) were diluted at 1:32 and 1:10 with Blood Bank Saline, respectively. The *anti-Dal* alloantibody was from a previously sensitized dog [University of Pennsylvania (11)], and monoclonal alloantibodies against *Kai 1* and *Kai 2* [Dr. He Young Kim, South Korea (12)] were used undiluted. We added 25 μl of the 2% RBC suspension and 25 μl of alloantibody reagent on top of the gel column. The mixture was incubated for 15 min at room temperature (~23°C), and then the gel column cards were centrifuged for 10 min at 85 x g in an ID-Centrifuge 6 S (DiaMed-ID, Microtyping System, DiaMed, Cressier, Switzerland). The results were then visually analyzed, and the results were graded from – to 4+ (Figure 3) depending on the location of the majority of RBCs within the gel column, as described (27, 29, 30).

### Statistical Analysis

Python v. 3.5.2 using Scipy^2^ v. 0.17.0 and Numpy v. 1.11.0 were used for statistical analyses. Chi-square test was done using Scipy's Chi-square implementation. Results were considered significant when *p* < 0.05. Additionally, Cohen's Kappa Test in Scikit v. 0.17.0 was used for comparison of *DEA 4* and *DEA 5* Card and Gel Tests.

## Results

In this survey, we screened 206 dogs from 18 breeds of six FCI groups, with ≥10 dogs for nine breeds (Table 1 and Supplement Table 1). They were 53.9% males and 43.7% females (2.4% unknown). The ages of the dogs ranged between 0.4 and 17 years (age unknown for nine dogs). Of the 64 (2^6^) possible blood type combinations, 14 different patterns were found, with two predominant patterns that only differed by *DEA 1* type; 47% of the dogs were *DEA 4*+*, DEA 5–, Dal*+*, Kai 1*+*, Kai 2–*, or *DEA 1*+, and 33% were *DEA 4*+*, DEA 5–, Dal*+*, Kai 1*+*, Kai 2–*, or *DEA 1–*; thus, 80% only differed by their *DEA 1* type. The other 12 observed blood type patterns were seen in only 0.5–5% of the dogs, without any specific breed association (patterns shown in Supplement Table 2).

**Table 1:** Typing results for blood groups *DEA 1, Dal, DEA 4, DEA 5, Kai 1*, and *Kai 2* in 206 dogs from Germany including breed and FCI^1^ group.

	**Dogs**		**Canine Blood Groups**							
**Breed (FCI^**1**^ Group)**	***N^**a**^***	***%^**b**^***	***DEA 1+ (%)***	***Dal+*** ***(%)***	***DEA 4+***	***DEA 5+***		***Kai 1+/*** ***Kai 2–***	***Kai 1–/*** ***Kai 2+***	***Kai 1–/*** ***Kai 2–***
					**Card^2^**	**Card**	**Gel**			
Cane Corso (2)	21	10.19	6 (28.6)^3^	18 (85.7)	21 (100)	2 (9.5)	2/15 (13.3)	21 (100)	0 (0)	0 (0)
Dalmatian (6)	21	10.19	20 (95.2)^3, 4^	18 (85.7)	21 (100)	4 (19.0)	2/14 (14.3)	21 (100)	0 (0)	0 (0)
Dobermann (2)	21	10.19	5 (23.8)^3^	16 (76.2)	21 (100)	5 (23.8)	6/13 (46.2)^5^	21 (100)	0 (0)	0 (0)
Labrador Retriever (8)	26	12.62	19 (73.1)^6^	26 (100)	26 (100)	0 (0)	0/20 (0)	26 (100)	0 (0)	0 (0)
Lhasa Apso (9)	21	10.19	12 (57.1)	18 (85.7)	21 (100)	2 (9.5)	2/18 (11.1)	16 (76.2)	5 (23.8)	0 (0)
Maltese (9)	28	13.59	13 (46.4)	28 (100)	28 (100)	6 (21.4)	5/23 (21.7)^5^	26 (92.9)	1 (3.6)	1 (3.6)
Pug (9)	20	9.71	20 (100)^3, 4^	18 (90.0)	20 (100)	0 (0)	0/20 (0)	20 (100)	0 (0)	0 (0)
Shih Tzu (9)	24	11.65	16 (66.7)	19 (79.2)	24 (100)	0 (0)	0/21 (0)	24 (100)	0 (0)	0 (0)
Boxer (2)	10	4.85	1 (10.0)^3^	10 (100)	10 (100)	0 (0)	1/8 (12.5)	10 (100)	0 (0)	0 (0)
9 breeds with <10 dogs^7^	14	6.79	10 (71.4)	13 (92.9)^8^	14 (100)	0 (0)	0/6 (0)	14 (100)	0 (0)	0 (0)
Total Number (N)	206		122	184	206	19	18^9^	199	6	1
%	100		59.2	89.3	100	9.2	11.4	96.6	2.9	0.5

1 Fédération Cynologique Internationale;

2 also all 158 dogs tested with Gel were DEA 4+;

3 correlation between breed and DEA 1 blood type was significant (p < 0.05);

4 significantly stronger reactions for DEA 1 were found;

5 correlation between breed and DEA 5 blood type was significant (p < 0.05);

6 significantly weaker/more moderate reactions for DEA 1 were found;

7 details are presented in Supplement Table 1;

8 1/1 Dal– Bullmastiff;

9 158 of 206 samples were DEA 5 typed by Gel;

*N^a^, Number of dogs; %^b^, % of all 206 dogs; DEA, Dog Erythrocyte Antigen*.

*Immunochromatographic strip, agglutination card, and gel column technique were used. Several blood group patterns were found (s. S.T. 2)*.

### *DEA 1* Typing Results

Utilizing the Strip with a monoclonal anti-*DEA 1* alloantibody, we were able to identify dogs with no reactivity, and thus *DEA 1–*, as well as dogs that were strongly positive, with *DEA 1* bands comparable to the positive control band. Of the 206 dogs, an equal proportion was either graded as *DEA 1–* (40.8%) or strongly *DEA 1*+ (40.3%) with a smaller proportion (18.9%) of weak to moderate *DEA 1*+ band reactions (Figure 1). Strong reactions (3+ and 4+) for *DEA 1* were found significantly more often in Dalmatians (*p* = 0.0003) and Pugs (*p* = 0.0003), and weak/moderate (1+/2+) reactions were significantly more common in Labrador Retrievers (*p* = 0.001). All Pugs and Dalmatians except for one Dalmatian were *DEA 1*+, and more Labrador retrievers and Shih Tzus were *DEA 1*+ than were other breeds (Table 1).

In contrast, less than a quarter of Cane Corsos and Doberman Pinschers were *DEA 1*+, and all but one Boxer was *DEA 1–* (Table 1). These breeds belong to the FCI group 2. A significant dependency between breed and *DEA 1* type was found for Cane Corso (*p* = 0.004), Dalmatian (*p* = 0.001), Doberman Pinscher (*p* = 0.001), Pug (*p* = 0.0002), and Boxer (*p* = 0.002).

### *DEA 4* Typing Results

When using the Card newly developed for *DEA 4* typing according to manufacturer's instructions, 9.2% of 206 dogs showed an extremely weak and thus questionable 1+ agglutination reaction, and 67.0% produced only weakly positive (1+/2+) results. However, when adding an additional 40 μl of diluent, all 191 tested dogs produced ≥1+ and clearly visible agglutination reactions, supportive of a *DEA 4*+ blood type. When comparing the results between the two diluent concentrations, 72% of the reactions stayed the same, 18% strengthened, and 10% became weaker (never weakening by more than one degree, e.g., 2+ became 1+), but none of the positive agglutination reactions turned negative (Supplement Table 3). Based on these results, the manufacturer's instructions have been changed to indicate that if the results using the initial 40 μl are unclear, another 40 μl of diluent should be added.

To further evaluate the results obtained by the new *DEA 4* typing card, we also applied the Gel using commercially available *anti-DEA 4* alloantibodies (antiserum; same as for the Card). As all dogs tested were *DEA 4*+ with the Card and Gel, we had perfect agreement (Kappa = 1) between the tests. Of the 158 dogs tested, all reacted strongly positively (3+/4+) and, therefore, were considered to have blood type *DEA 4*+ (Figure 3). While all agglutination reactions observed by the Gel were 3+ to 4+, such strong reactions (3+ to 4+) using Cards were only seen in 23.8 and 15.7% using 40 and 80 μl of diluent, respectively. While the regression of *DEA 4* agglutination strength with 40 μl and 80 μl in the 191 tested dogs showed a good correlation (*r* = 0.8999), there was no significant correlation of strength between the Gel and Card (*r* = 0.0754). Also, the degree of *DEA 4* positivity did not appear to be associated with any particular breed.

### *DEA 5* Typing Results

When using the new *DEA 5* typing card according to the manufacturer's instructions, 90.8% of 206 dogs did not show any RBC-agglutination reactions. Weak agglutination reactions (1+ or 2+) were seen in 19 dogs, and they were thus considered *DEA 5*+ (Table 1). The manufacturer states that the *DEA 5* agglutination is “finer and less plentiful than that observed in a *DEA 1* dog run on a *DEA 1* test card” (DMS) and we also found the *DEA 5*+ card agglutination results to be consistently less intense than the *DEA 4*+ card results. Utilizing the Gel with the same polyclonal *DEA 5* antibody used for the Card, 11.4% of the 158 dogs typed *DEA 5*+. With the Gel, six (33.3%) of the *DEA 5*+ dogs reacted only weakly/moderately positive, while the other 12 *DEA 5*+ dogs reacted strongly (3+/4+).

When comparing the Card and Gel methods, 97% of the *DEA 5* results were concordant; in four of five discordant results, the Gel test indicated *DEA 5*+ (Table 2). The regression of *DEA 5* Gel and Card strength in all 158 tested dogs showed a good correlation (*r* = 0.8329), and the two methods showed almost perfect agreement (Kappa = 0.831). For the Doberman Pinscher (*p* = 0.02) and Maltese (*p* = 0.03), the *DEA 5*+ blood type was significantly associated with the breed.

**Table 2:** Comparison and results for *DEA 5* Card and Gel for 158 dogs typed by both methods.

***N* = 158**	***Gel DEA 5*** **Typing Results**	
	***DEA 5+***	***DEA 5–***
Card *DEA 5* typing results *DEA 5*+	14	1
*DEA 5*–	4	139
Gel *DEA 5* typing results N (%)	18/11.39%	140/88.61%
Card *DEA 5* typing results N (%)	15/9.49%	143/90.51%

Using the Card, 9% of all 206 dogs and also 9% of the 158 dogs that were typed by both Card and Gel were *DEA 5*+, while 11% of those 158 dogs were *DEA 5*+ using the Gel. *DEA 5*+ dogs were found by the Card and Gel in Cane Corso, Dalmatian, Doberman, Lhasa Apso, and Maltese, and by the Gel also in one Boxer (Table 1).

### *Dal* Typing Results

Strong (3+/4+) agglutination reactions for *Dal* using a polyclonal anti-*Dal* antiserum in a Gel assay were observed in 183 of the *Dal*+ dogs, and a 2+ reaction was seen in one Shih Tzu that was on immunosuppressive therapy. In addition to the previously reported breeds with *Dal–* dogs, we found 22 *Dal–* dogs also in the breeds Cane Corso, Pug, and Mastiff (Table 1). We did not find any significant association between breed and *Dal* type, but the number of dogs per breed is small.

### *Kai 1* and *Kai 2* Typing Results

**Figure 4:**
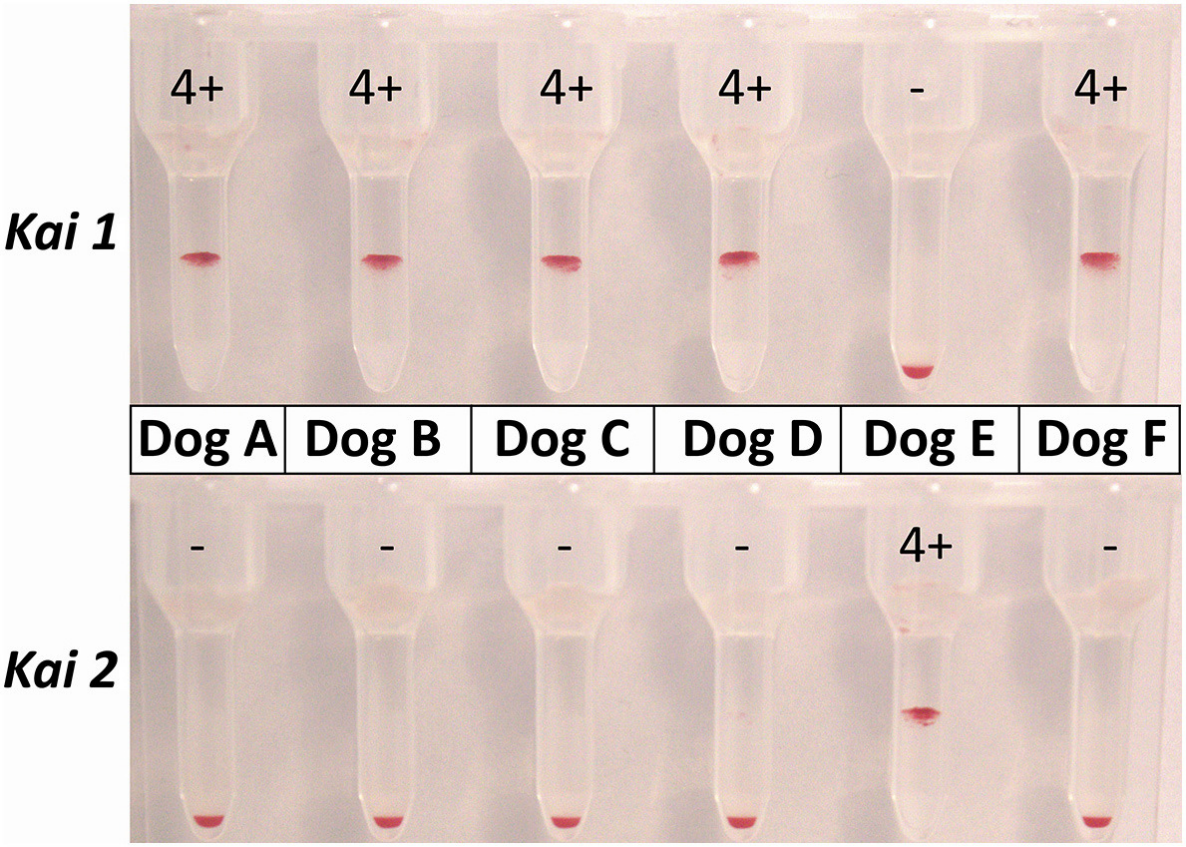
Gel column typing results for the same six dogs for *Kai 1* and *Kai 2*. Dogs A–D and F are showing the most common *Kai 1*+*/Kai 2–* typing pattern, while Dog E has a *Kai 1–/Kai 2*+ pattern.

Utilization of the Gel and specific monoclonal antibodies against *Kai 1* and *Kai 2* resulted in either very strong agglutination reactions or completely negative test results (Figure 4). Overall, 96.6% of all dogs were *Kai 1*+*/Kai 2–* (Table 3). There were only five Lhasa Apsos and one Maltese that were *Kai 1–/Kai 2*+. Furthermore, only a single Maltese dog was found to be *Kai 1–/Kai 2–* in this survey (Table 1).

**Table 3:** Results for *Kai 1* and *Kai 2* typing of 206 dogs typed by the gel column method.

**Blood type**	***Kai 1+***	***Kai 1–***
	**N (%)**	**N (%)**
*Kai 2+*	0 (0)	6 (2.9)
*Kai 2–*	199 (96.6)	1 (0.5)

## Discussion

Blood transfusions are pivotal in the supportive care of anemic and bleeding dogs, but the recruitment of blood donors and assuring blood type compatibility pose challenges. While *DEA 1* typing is well established, limited availability of other typing reagents, in-practice kits, and laboratory methods have hampered the extended blood typing of dogs. In this comprehensive blood typing survey of 206 dogs from Germany, we used established and introduced new typing methods for several canine blood types and found significant differences in the prevalence of specific blood types among certain breeds. Overall, 80% of all dogs were *DEA 4*+*, DEA 5–, Dal*+, and *Kai 1*+*/2–* but could be either *DEA 1*+ or *DEA 1–*. While the Gel and Strip are the most accurate standardized methods, the new *DEA 4* and *DEA 5* typing cards are welcome diagnostic kits to extend the typing repertoire in clinical practice and assist with the investigation and prevention of blood incompatibilities.

### *DEA 1* Typing

Overall, 40.8 % of all dogs in our survey were *DEA 1–*, which is very similar to recent surveys from USA, France, India, and Italy that also utilized the same immunochromatographic method and monoclonal *anti-DEA* 1 alloantibody (25, 27, 31, 37, 40). Comparison to prior studies is complicated, because these studies utilized polyclonal alloantibodies against *DEA 1.1* and *DEA 1.2* (7). However, recent studies clearly showed a close association between *DEA 1* typing and *DEA 1.1* typing results (19). In fact, the original monoclonal *anti-DEA 1.1* alloantibodies used in *DEA 1* typing kits were later renamed as *DEA 1* by the manufacturers. The relationship between *DEA 1* and *DEA 1.2* is less clear, but it is likely that *DEA 1.2* is a weaker *DEA 1* antigen (20). No laboratories currently offer *DEA 1.2* typing or any reagents for *DEA 1.2* typing. Nevertheless, prior surveys using polyclonal *anti-DEA 1.1* and *anti-DEA 1.2* antisera also showed a predominance of *DEA 1*+ dogs (Table 4). A comparative overview of prior *DEA 1* typing surveys is shown in Table 4.

**Table 4:** Comparative table for the current survey and previously reported *DEA 1* blood type prevalence per region.

**Number of Dogs**	***DEA 1+* (%)**	**Region**	**Reference**	**Method**
206*	59.2	Germany	This study	Strip^1^
66	69.7	North America	(19)	Strip^1^
88	55	North America	(24)	Gel^4^
1037	62	Italy	(25)	Strip^1^
178	65.2	Turkey	(26)	Gel^4^
503	59.6	North America	(27)	Strip^1^
274	56.9	Portugal	(28)	Gel^4^
43	53.5	North America	(30)	Gel^4*^
7414	61.2	Italy	(31)	Strip^1^, Card^2^
206	53.4	Spain	(32)	Cartridge^3^
304	53	Switzerland	(33)	Gel^4^
92	75	Spain (Ibiza)	(34)	Card^2^
233	47	South Africa	(35)	Card^2^
80/79	78.7^1^/57.0^1^	France/North America	(37)	Strip^1^
23	56.5^1^/39^2^	North America	(39)	Card^1^, Gel^2^, *
125	61.6	India	(40)	Strip1
100	78	South Africa	(41)	Strip^1^

1 Canine Quick Test/Lab Test BT DEA 1, Alvedia

2 Card RapidVet-H Canine DEA 1.1, DMS Laboratories

3 QuickTest DEA 1.1, Alvedia

4 ID- Gel Test DEA 1.1, DiaMed (*other methods not shown); DEA—Dog Erythrocyte Antigen.

Far more (71–90%) of the tested Cane Corsos, Doberman Pinschers, and Boxers were *DEA 1–*. Interestingly, these breeds belong to the FCI group 2, while other breeds studied were in other groups. Boxers and Cane Corso from various other European countries and South Africa were also mostly *DEA 1–* in prior surveys (details in Supplement Table 4). Thus, these breeds may be preferentially considered when recruiting blood donors to increase the *DEA 1–* donor pool, as suggested previously (25, 42)

In contrast, the Dalmatians and Pugs in our survey were all *DEA 1*+, except for one Dalmatian. Also, the Labrador Retriever and Shih Tzu breeds had mostly *DEA 1*+ dogs. The predominance of *DEA 1*+ dogs in these breeds was also observed in recent surveys from Switzerland, South Africa, Portugal, and India (details in Supplement Table 4). Finally, the Maltese breed had a near equal proportion of *DEA 1*+ and *DEA 1–* dogs, as previously reported from another survey in Italy (31). Thus, these *DEA 1* typing surveys show similar proportions of *DEA 1*+ and *DEA 1–* dogs in each breed worldwide, which is not surprising considering their common ancestors and international breeding practices within breeds.

The strength of *DEA 1* expression in *DEA 1*+ dogs is similar to a prior survey from the USA (27). Equal proportions are either strongly *DEA 1*+ or *DEA 1–*. Only a fifth of dogs show weak or moderate *DEA 1* expression, an important observation for clinical practice, as the latter may lead to difficulties in interpretation and, potentially, in sensitizing dogs. Indeed, even blood from a weakly *DEA 1*+ donor given to a *DEA 1–* dog can elicit the sustained production of *anti-DEA 1* alloantibodies (38), and sensitized dogs may develop an acute hemolytic transfusion reactions when again transfused (14).

### *DEA 4* Typing

All dogs in this survey were typed as *DEA 4*+ by the Gel and Card. Only a few prior typing surveys included *DEA 4* typing, and these studies also revealed that almost all dogs are *DEA 4*+, as *DEA 4–* dogs are extremely rarely observed (7, 16, 27, 30, 37, 39). Thus, while *DEA 4* is known as a blood group system, it should be considered a high-frequency antigen, as less than 1% of dogs are *DEA 4–* (14, 43).

The new *DEA 4* typing card test was easy to perform, but some results were challenging to interpret. Agglutination reactions on the Card were weaker than those observed with the Control on the Card or with the Gel. The addition of another 40 μl diluent enhanced the weak agglutination reactions, and this phenomenon might be caused by a Prozone-like effect, though this was not further studied. The varying degrees of agglutination reactions observed with the Cards remain unexplained but are likely technical, as all 158 dogs tested produced strongly positive (3+ to 4+) results by the Gel method. Optimization of the Card to increase agglutination reactions would enhance the reliability of this assay. In the meantime, the manufacturer has independently adjusted the protocol based upon our observations.

Because the instructions are identical to *DEA 1* typing cards, the *DEA 4* cards can be readily added to the typing repertoire in clinical practice. The *DEA 4* typing cards may be particularly useful for clinical diagnostics of incompatibility reactions in previously transfused *DEA 4–* dogs and for identifying *DEA 4–* donors. Indeed, early experimental studies (8) reported blood incompatibilities caused by *DEA 4*+ alloantibodies, and there is one clinical case report of an acute hemolytic transfusion reaction after the dog had been sensitized by a prior *DEA 4*+ transfusion (4). Thus, identifying *DEA 4–* donors could be very valuable when facing broad blood incompatibilities in previously transfused dogs potentially caused by *DEA 4* sensitization.

### *DEA 5* Typing

In this survey from Germany, we found 9 and 11% *DEA 5*+ dogs with the Card and Gel, respectively. Similarly, a limited survey from the Netherlands identified 17% *DEA 5*+ (44), and other surveys from North America found 12–25% *DEA 5*+ dogs (14, 16). Although *DEA 5* has been associated with blood type incompatibilities and reduced RBC lifespan experimentally (8), there are no clinical reports of any acute hemolytic transfusion reactions related to *DEA 5*. The new *DEA 5* typing cards will be useful in the practical diagnostic examination of blood type incompatibilities and acute hemolytic transfusion reactions in previously transfused dogs.

Finally, the new commercially available *DEA 4* and *DEA 5* typing cards used in this survey produced similar but weaker agglutination results to the gel column method. Moreover, the agglutinations were weaker than seen with the commonly used *DEA 1* cards. Nevertheless, there was a strong correlation between the two methods, with only five discordant *DEA 5* typing results, supporting their use in clinical settings.

### *Dal* Typing

While original reports suggested that the *Dal–* blood type was exclusive to Dalmatians (11), recent studies from North America also found *Dal–* dogs in other breeds (27, 29, 36). Similarly, our survey from Germany found 10.7% *Dal–* dogs. This includes dogs from breeds with previously described *Dal–* dogs like Dalmatians, Dobermans, Shih Tzu, and Lhasa Apso (27, 29, 36), and also, for the first time, in Cane Corsos, Pugs, and a Mastiff. The prevalence of *Dal–* dogs in specific breeds is difficult to compare, because of the small numbers of dogs in each breed typed and reported geographical differences in the prevalence of *Dal* *(**29**)*. Clearly, *Dal* typing is warranted for those breeds as well as for additional breeds, as *Dal* type blood incompatibility could potentially lead to an acute hemolytic transfusion reaction (29). Thus, it would be beneficial to identify *Dal–* donors for use in *Dal–* patients. Unfortunately, at this point, there is no commercial *Dal* typing kit available.

While the Gel uniformly showed strong (3+/4+) agglutination reactions with *Dal*+ blood, there was one dog that was receiving long-term treatment with immunosuppressives that had only a 2+ reaction. It may be possible that immunosuppression reduces the expression of the *Dal* antigen or the binding with the antibody. Differences in antigen expression have been shown in humans with illnesses (45) and were suspected in FeLV+ cats (46).

### *Kai 1* and *Kai 2* Typing

Utilizing the Gel, the agglutination reactions were very strong for *Kai 1* and *Kai 2*. In this survey, 96.6% of all dogs were *Kai 1*+*/Kai 2–*, and few dogs were *Kai 1–* and *Kai 2*+ or *Kai 2–*. Thus, our results for the *Kai 1 /Kai 2* blood type pattern distribution were similar to a prior survey from North America, which found 94% *Kai 1*+*/Kai 2–, 5% Kai 1–/Kai 2–*, and 1% *Kai 1–/Kai 2*+ (27). In a recent survey using the conventional tube agglutination assay with 203 mostly Mastiff dogs from South Korea, the proportion of *Kai 1*+*/Kai 2–* was only 42%, with the proportion of *Kai 1–/Kai 2*+ being 37% and that of *Kai 1–/Kai 2–* being 20% (12). The large group of *Kai 1–/Kai 2*+ Mastiffs seen in Korea could not be substantiated here and in the survey from North America because of a lack of samples received from this breed. Noteworthy, however, is the absence of dogs that were *Kai 1*+ as well as *Kai 2*+. While the *Kai 1* and *Kai 2* antigens appear to be very different based on protein size (12), it cannot be excluded that these two antigens are genetically related.

Finally, several Lhasa Apsos were found to be *Kai 1– /Kai 2*+ in the United Kingdom (Watson et al., personal communication). Because the only other *Kai 1–/Kai 2*+ dog was a Maltese and another Maltese was *Kai 1–/Kai 2–*, it is possible that these two related breeds of the FCI Group 9 are unique regarding their *Kai* antigen expressions.

However, the clinical importance of the *Kai 1* and *Kai 2* blood groups needs to be further investigated, especially since the development of alloantibodies against *Kai 1* and *Kai 2* in previously transfused dogs has been reported (12).

## Limitations

Although this is the first published survey for *Dal, Kai 1*, and *Kai 2* in Europe, it was primarily limited to Germany geographically. While over 200 dogs were typed for many blood types, the reported blood type prevalences for each breed are still limited by sample size. The prevalences could well be impacted if more dogs are typed and/or if dogs from other geographic regions are included. Because several reagents for typing are not readily available, and the clinical importance of some of the blood types tested has not been established, larger surveys are unlikely to be done.

This study was performed under strict laboratory conditions in a reference diagnostic laboratory, with the same trained personnel (AE, SF) performing all the tests and with appropriate control samples. Typing conditions in private practice are likely to be less favorable, and weak agglutination and binding reactions will be difficult to interpret. When uncertain, blood samples should be sent to a reference laboratory for retyping and potentially back-typing if possible.

Notably, this study did not evaluate the presence of naturally occurring alloantibodies in any dogs. However, the presence of naturally occurring alloantibodies has been evaluated in prior studies (12, 15, 16, 27, 29, 30), and they do not appear to cause acute hemolytic transfusion reactions when transfusing a dog for the first time (1, 8). Specific alloantibody studies of plasma or serum from previously transfused dogs, which were extensively typed as in this survey, would be of interest, although only rarely is a known RBC antigen identified to cause a hemolytic transfusion reaction (3, 4, 14).

This study excluded typing for *DEA 7*, because commercial reagents were not available at the time this survey was performed, and in the past, the available typing reagents have given *DEA 7* typing and alloantibody results that have been difficult to reproduce (27). Therefore, the presence of naturally occurring *anti-DEA* 7 alloantibodies and their importance in eliciting an acute hemolytic transfusion reaction independent of whether a dog was previously transfused or not remains unclear (7, 8, 14, 15, 42, 43).

## Conclusions

The options for extended in-house typing, including the two now commercially available *DEA 4* and *DEA 5* cards, may be especially useful in previously transfused dogs, as well as in blood incompatibility work-up in a practice setting. While in-house typing reduces the turnaround time, tests should always be performed by trained personnel and, in the case of uncertain results, retyping and back-typing should be performed in a reference laboratory. Additional typing for *Dal* and *Kai 1/2* with the Gel method should remain restricted to reference laboratories for now.

## Data Availability Statement

The datasets generated for this study are available on request to the corresponding author.

## Ethics Statement

Ethical review and approval was not required for the animal study because only leftover EDTA blood samples sent for routine diagnostic work were used. Approval for further use of surplus material is given when submitting to Laboklin as stated in the Laboklin policy.

## Author Contributions

AE performed typing, data analysis, and manuscript writing. SF performed tests and sample preparation. CW gathered samples, provided demographic data, and reviewed the manuscript. EM contributed to the concept, funding, material acquisition, and manuscript review. UG contributed to the concept of the study, material acquisition, data analysis, and manuscript writing.

### Conflict of Interest

The authors declare that they are associated with either the Laboklin GmbH ' Co KG, Kissingen, Germany or PennGen Laboratory, University of Pennsylvania, Philadelphia, PA, USA. Both offer blood typing services. This survey is part of AE's doctoral thesis at the University of Zürich, Switzerland, with UG being her mentor. Laboklin provided the workplace and doctoral fellowship of AE and was involved in the study; the study received funding from Laboklin and was executed at Laboklin GmbH ' Co KG, Kissingen and Radolfzell, Germany, but was performed independently from Laboklin's routine laboratory work.

## Footnotes

^1^http://www.fci.be/en/

^2^https://www.scipy.org

## Abbreviations

Card: agglutination card (RapidVet-H *DEA 4* Agglutination Card Test and *DEA 5* Card RapidVet-H *DEA 5* Agglutination Card Test, DMS, Flemington, NJ, US)
*DEA*: dog erythrocyte antigen
EDTA: ethylene-diaminetetraacetic acid
FCI: Federation Cynologique Internationale
Gel: gel column method (BioRad ID-Cards, NaCl, Enzyme Test and Cold Agglutinins, DiaMed)
RBC: red blood cell
Strip: immunochromatographic strip technique (Alvedia, Lab Test *DEA 1*, Limonest, France).
