# Supplementary material for: Survey of Blood Groups DEA 1, DEA 4, DEA 5, Dal, and Kai 1/Kai 2 in Different Canine Breeds From a Diagnostic Laboratory in Germany
Source: Front Vet Sci. 2020 Feb 28;7:85. doi: 10.3389/fvets.2020.00085 (PMC7058700; doi:10.3389/fvets.2020.00085)
Supplement: Supplementary file 1 [file Data_Sheet_1.DOCX]

**Supplement Table 1 Typing results from 9 additional breeds with <10 dogs.** These 14 dogs were summarized as ‘9 breeds with <10 dogs’ in Table 1

| ***Breed ( FCI^1^ Group)*** | ***Number of dogs*** | | ***% of all dogs tested*** | ***DEA 1+*** | ***Dal+*** | ***DEA 4+* Card** | ***Kai 1+/***  ***Kai 2-*** |  |
| --- | --- | --- | --- | --- | --- | --- | --- | --- |
| Bergamasco Shepherd Dog (1) | 1 | | 0.49 | 1 | 0 | 1 | 1 |  |
| Bullmastiff (2) | 1 | | 0.49 | 1 | 1 | 1 | 1 |  |
| Chihuahua (9) | 3 | | 1.46 | 3 | 0 | 3 | 3 |  |
| Coton de Tulear (9) | 1 | | 0.49 | 1 | 0 | 1 | 1 |  |
| Flat Coated Retriever (8) | 1 | | 0.49 | 0 | 0 | 1 | 1 |  |
| Giant Schnauzer (2) | 2 | | 0.97 | 2 | 0 | 2 | 2 |  |
| Havanese (9) | 2 | | 0.97 | 1 | 0 | 2 | 2 |  |
| Malinois (1) | 2 | | 0.97 | 1 | 0 | 2 | 2 |  |
| Staffordshire Bullterrier (3) | 1 | | 0.49 | 0 | 0 | 1 | 1 |  |
| Total | 14 | 6.8 | | 10 | 1 | 14 | 14 |  |
| % | 100 |  |  | 71.4 | 7.1 | 100 | 100 |  |
| There were no *DEA 5+, Kai 1-/Kai 2+, Kai 1-/Kai 2-* dogs.  All dogs tested were also *DEA 4+* with gel column method. | | | | | | | | |
| ^1^Fédération Cynologique Internationale; *DEA* Dog Erythrocyte Antigen | | | | | | | | |
