# Supplementary material for: Survey of Blood Groups DEA 1, DEA 4, DEA 5, Dal, and Kai 1/Kai 2 in Different Canine Breeds From a Diagnostic Laboratory in Germany
Source: Front Vet Sci. 2020 Feb 28;7:85. doi: 10.3389/fvets.2020.00085 (PMC7058700; doi:10.3389/fvets.2020.00085)
Supplement: Supplementary file 2 [file Data_Sheet_2.DOCX]

**Supplement Table 2 Prevalence (%) for blood type patterns from 206 dogs for blood types *DEA 1, DEA 4, DEA 5, Dal, Kai 1* and *Kai 2*.**

| ***Blood Type*** | | | | | |  |
| --- | --- | --- | --- | --- | --- | --- |
| ***DEA 1*** | ***DEA 4*** | ***DEA 5*** | ***Dal*** | ***Kai 1*** | ***Kai 2*** | **% of dogs showing that pattern** |
| + | + | - | + | + | - | 47.09 |
| - | + | - | + | + | - | 33.50 |
| + | + | - | - | + | - | 4.85 |
| + | + | + | + | + | - | 3.88 |
| - | + | - | - | + | - | 3.40 |
| - | + | + | + | + | - | 2.43 |
| + | + | - | + | - | + | 0.97 |
| + | + | + | - | + | - | 0.97 |
| - | + | - | - | - | + | 0.49 |
| - | + | + | - | + | - | 0.49 |
| - | + | + | + | - | + | 0.49 |
| + | + | - | - | - | + | 0.49 |
| + | + | + | + | - | - | 0.49 |
| + | + | + | + | - | + | 0.49 |
| + positive for tested blood type; | | | | | | |
| - negative for tested blood type. *DEA* Dog Erythrocyte Antigen | | | | | | |
