# Supplementary material for: Survey of Blood Groups DEA 1, DEA 4, DEA 5, Dal, and Kai 1/Kai 2 in Different Canine Breeds From a Diagnostic Laboratory in Germany
Source: Front Vet Sci. 2020 Feb 28;7:85. doi: 10.3389/fvets.2020.00085 (PMC7058700; doi:10.3389/fvets.2020.00085)
Supplement: Supplementary file 3 [file Data_Sheet_3.DOCX]

**Supplement Table 3 Comparison of the agglutination strength on *DEA 4* agglutination cards depending on using 40 µl or 80 µl buffer.** When adding another 40 µl buffer to the initial 40 µl buffer used for the *DEA 4* cards, the intensity did not change in most reactions, became stronger by 1 to 2 grades in 18% and 1 degree less in 10% of the reactions of the 191 samples tested. It is important to note, that reactions that were very weak turned clearly positive when using 80 µl instead of 40 µl buffer.

| **Reaction intensity with 80 µl compared to 40 µl buffer** | **N** | **%** |
| --- | --- | --- |
| Stronger | 35 | 18.3 |
| Same | 137 | 71.7 |
| Weaker | 19 | 10.0 |
