# Supplementary material for: Survey of Blood Groups DEA 1, DEA 4, DEA 5, Dal, and Kai 1/Kai 2 in Different Canine Breeds From a Diagnostic Laboratory in Germany
Source: Front Vet Sci. 2020 Feb 28;7:85. doi: 10.3389/fvets.2020.00085 (PMC7058700; doi:10.3389/fvets.2020.00085)
Supplement: Supplementary file 4 [file Table_4.docx]

**Supplement Table 4 Comparative table of *DEA 1* prevalence in previous studies in breeds with ≥10 dogs in the current study per region.**

| **Breed** | **Number of dogs** | ***DEA 1+*** | **Region** | **Reference** | **Methods** |
| --- | --- | --- | --- | --- | --- |
| **Boxer** | 10 | 10% | Germany | This Survey | Strip^1^ |
|  | 22 | 18% | Italy | (25) | Strip^1^ |
|  | 13 | 0% | Portugal | (28) | Gel^3^ |
|  | 106 | 17% | Italy | (31) | Strip^1^,Card^2^ |
|  | 8 | 0% | Switzerland | (33) | Gel^3^ |
|  | 8 | 13% | South Africa | (35) | Card^2^ |
| **Cane Corso** | 21 | 29% | Germany | This Survey | Strip^1^ |
|  | 31 | 32% | Italy | (25) | Strip^1^ |
|  | 76 | 50% | Italy | (31) | Strip^1^,Card^2^ |
|  | 46 | 29% | Italy | (41) | Strip^1^ |
| **Dalmatian** | 21 | 95% | Germany | This Survey | Strip^1^ |
|  | 4 | 75% | Switzerland | (33) | Gel^3^ |
|  | 4 | 100% | South Africa | (35) | Card^2^ |
|  | 6 | 50% | India | (40) | Strip^1^ |
| **Doberman** | 21 | 24% | Germany | This Survey | Strip^1^ |
|  | 17 | 6% | Italy | (25) | Strip^1^ |
|  | 12 | 0% | Portugal | (28) | Gel^3^ |
|  | 6 | 33% | Switzerland | (33) | Gel^3^ |
|  | 5 | 60% | South Africa | (35) | Card^2^ |
|  | 10 | 80% | India | (40) | Strip^1^ |
| **Labrador Retriever** | 26 | 73% | Germany | This Survey | Strip^1^ |
|  | 69 | 77% | Italy | (25) | Strip^1^ |
|  | 29 | 45% | Portugal | (28) | Gel^3^ |
|  | 478 | 65% | Italy | (31) | Strip^1^,Card^2^ |
|  | 20 | 75% | Switzerland | (33) | Gel^3^ |
|  | 20 | 55% | South Africa | (35) | Card^2^ |
|  | 44 | 59% | India | (40) | Strip^1^ |
| **Maltese** | 28 | 46% | Germany | This Survey | Strip^1^ |
|  | 4 | 75% | Italy | (25) | Strip^1^ |
|  | 130 | 55% | Italy | (31) | Strip^1^,Card^2^ |
|  | 2 | 50% | Switzerland | (33) | Gel^3^ |
| **Pug** | 20 | 100% | Germany | This Survey | Strip^1^ |
|  | 4 | 100% | Italy | (25) | Strip^1^ |
|  | 61 | 77% | Italy | (31) | Strip^1^,Card^2^ |
| **Shih Tzu** | 24 | 67% | Germany | This Survey | Strip^1^ |
|  | 79 | 72% | Italy | (31) | Strip^1^,Card^2^ |

^1^Canine Quick Test/Lab Test BT DEA 1, Alvedia , ^2^Card RapidVet-H Canine DEA 1.1, DMS Laboratories

^3^ID- Gel Test DEA 1.1, DiaMed DEA Dog Erythrocyte Antigen
